# Supplementary material for: Influence of Additional White, Red and Far-Red Light on Growth, Secondary Metabolites and Expression of Hormone Signaling Genes in Scots Pine under Sunlight
Source: Cells. 2024 Jan 19;13(2):194. doi: 10.3390/cells13020194 (PMC10813845; doi:10.3390/cells13020194)
Supplement: Supplementary file 1 [file cells-13-00194-s001.zip › cells-2765139-supplementary.pdf]

Table S1. Primers for genes

№	Gene Bank ID	Gene Description	Gene	Primer 5′-3′	
				forward	reverse
1	ALN42232.1	Histidine-containing phosphotransfer 1	*HPT1*	GCTCAAGTATAGGAGCGCGG	CCAGCTTGTTTTTCACGAGGT
2	EF083399.1	Jasmonate-Zim domain 1	*JAZa*	GGTGAACGTGTATGATGATATTCC	CGTTGCAGAGAATGCTTCCTC
3	AY289600.1	Auxin-induced protein 1 (IAA1)	*AUX/IAA*	GCCACCTGTCAAAGATTTCAG	TGAGGTCCACCTTTCTGAGA
4	CBB44933.1	Actin 1	*ACT1*	TTAGCAACTGGGATGACATGGA	CCTGAATGGCAACATACATAGCA
5	KY914544.1	auxin response factor 16	*ARF16*	TATATACCGTGGCACACCGC	ACACACAGCTCCCCATTGAG
6	A9NWW4	Steroid 5-α-reductase DET2	*BR–α–RED*	CAACAGAGCTCTCAAGGCGA	GAAGAGTAGGGCGCTCATCC
7	MH017214.1	Brassinosteroid mediated signaling pathway gene	*BRZ2*	GCGTCTATGAGCCCATCCTC	CGGCGAGATTTTTCAGCCAG
8	JQ240296.1	Monoterpene synthase like TPS–mono1	*mono-TERP*	GGAGTATCCACCAGTCCCCT	TCCAATGCCTTTCTCGGTCC
9	JQ240308.1	α-terpineol synthase TPS–α-terp	*α-TERP*	AGCATCGAACGTTTGGGAGT	AAGAGTTCGAAGGCCCAGTG
10	MA_10426264g0020	Chalcone synthase	*CHS*	GGCATTCAGGAAGGCTCAGAGA	GGCACCTCCACCACAACCAT
11	FN433184.1	Auxin response factor 3/4	*ARF3/4*	CGTGGCTCCTTCCTCAACAT	TGGTCGGCGTGTAGATTGAC
12	L26923.1	Glyceraldehyde–phosphate dehydrogenase	*GAPDH*	GACCCAGATGTGCAGGTAGC	GTCAACAAACACGCCAGGTC

Table S2. Gene expression of Scots pine saplings

Genes	Control	Additional light			
		WL	RL	FRL	RL+FRL
**current-year needles**					
ARF16	1.00 ± 0.00 ^{d}	0.90 ± 0.05 ^{d}	4.72 ± 0.10 ^{c}	4.98 ± 0.12 ^{b}	9.01 ± 0.09 ^{a}
AUX/IAA	1.00 ± 0.00 ^{c}	0.66 ± 0.02 ^{d}	4.07 ± 0.46 ^{b}	0.61 ± 0.02 ^{d}	8.12 ± 0.10 ^{a}
JAZa	1.00 ± 0.00 ^{d}	1.35 ± 0.08 ^{c}	6.21 ± 0.55 ^{b}	0.10 ± 0.03 ^{e}	8.74 ± 0.50 ^{a}
HPT1	1.00 ± 0.00 ^{b}	1.32 ± 0.10 ^{ab}	0.88 ± 0.05 ^{b}	1.08 ± 0.08 ^{b}	1.50 ± 0.13 ^{a}
BR-a-RED	1.00 ± 0.00 ^{c}	1.66 ± 0.13 ^{c}	5.56 ± 0.71 ^{b}	5.55 ± 0.42 ^{b}	8.26 ± 0.62 ^{a}
BRZ2	1.00 ± 0.00 ^{b}	0.48 ± 0.19 ^{b}	0.92 ± 0.26 ^{b}	0.71 ± 0.21 ^{b}	5.19 ± 0.14 ^{a}
mono-TERP	1.00 ± 0.00 ^{d}	3.11 ± 0.22 ^{c}	4.89 ± 0.28 ^{b}	1.47 ± 0.12 ^{d}	8.56 ± 0.47 ^{a}
α-TERP	1.00 ± 0.00 ^{c}	0.62 ± 0.08 ^{c}	0.47 ± 0.06 ^{c}	3.64 ± 0.27 ^{b}	8.28 ± 0.42 ^{a}
CHS	1.00 ± 0.00 ^{c}	0.23 ± 0.02 ^{d}	1.62 ± 0.13 ^{b}	0.28 ± 0.02 ^{d}	8.61 ± 0.56 ^{a}
**cambium of previous-year shoot**					
ARF16	1.00 ± 0.00 ^{b}	0.80 ± 0.33 ^{b}	0.68 ± 0.30 ^{b}	1.95 ± 0.35 ^{b}	5.03 ± 0.09 ^{a}
AUX/IAA	1.00 ± 0.00 ^{b}	1.82 ± 0.37 ^{b}	2.29 ± 0.31 ^{ab}	2.42 ± 0.48 ^{ab}	3.40 ± 0.47 ^{a}
JAZa	1.00 ± 0.00 ^{d}	4.65 ± 0.38 ^{b}	2.68 ± 0.41 ^{c}	0.99 ± 0.18 ^{d}	8.33 ± 0.60 ^{a}
HPT1	1.00 ± 0.00 ^{a}	1.03 ± 0.17 ^{a}	1.25 ± 0.14 ^{a}	1.17 ± 0.31 ^{a}	1.55 ± 0.19 ^{a}
BR-a-RED	1.00 ± 0.00 ^{c}	0.61 ± 0.04 ^{e}	0.81 ± 0.06 ^{d}	2.75 ± 0.42 ^{b}	7.09 ± 0.79 ^{a}
BRZ2	1.00 ± 0.00 ^{b}	0.47 ± 0.11 ^{b}	2.72 ± 0.28 ^{a}	2.23 ± 0.26 ^{a}	2.61 ± 0.28 ^{a}
mono-TERP	1.00 ± 0.00 ^{a}	0.55 ± 0.34 ^{a}	0.38 ± 0.16 ^{a}	0.44 ± 0.25 ^{a}	0.87 ± 0.29 ^{a}
α-TERP	1.00 ± 0.00 ^{a}	0.40 ± 0.04 ^{a}	0.20 ± 0.04 ^{a}	0.48 ± 0.20 ^{a}	0.56 ± 0.30 ^{a}
CHS	1.00 ± 0.00 ^{a}	0.85 ± 0.08 ^{ab}	0.56 ± 0.06 ^{b}	0.26 ± 0.05 ^{b}	0.47 ± 0.21 ^{b}
ARF 3/4	1.00 ± 0.00 ^{d}	2.17 ± 0.31 ^{c}	2.93 ± 0.28 ^{c}	4.41 ± 0.32 ^{b}	6.73 ± 0.52 ^{a}
**young roots**					
ARF16	1.00 ± 0.00 ^{b}	2.30 ± 0.18 ^{b}	2.19 ± 0.14 ^{b}	6.23 ± 0.50 ^{a}	7.92 ± 0.60 ^{a}
AUX/IAA	1.00 ± 0.00 ^{c}	1.12 ± 0.11 ^{c}	1.05 ± 0.14 ^{c}	8.65 ± 0.23 ^{a}	7.86 ± 0.43 ^{b}
JAZa	1.00 ± 0.00 ^{a}	1.31 ± 0.18 ^{a}	1.19 ± 0.10 ^{a}	1.04 ± 0.11 ^{a}	0.98 ± 0.07 ^{a}
HPT1	1.00 ± 0.00 ^{c}	1.69 ± 0.24 ^{c}	6.16 ± 0.61 ^{b}	4.63 ± 0.36 ^{b}	9.02 ± 0.29 ^{a}
BR-a-RED	1.00 ± 0.00 ^{d}	1.29 ± 0.10 ^{d}	2.16 ± 0.20 ^{c}	2.93 ± 0.19 ^{b}	6.14 ± 0.45 ^{a}
BRZ2	1.00 ± 0.00 ^{d}	1.65 ± 0.18 ^{cd}	2.18 ± 0.08 ^{c}	3.83 ± 0.28 ^{b}	8.20 ± 0.54 ^{a}
mono-TERP	1.00 ± 0.00 ^{b}	0.93 ± 0.14 ^{b}	1.29 ± 0.18 ^{b}	1.12 ± 0.21 ^{b}	3.12 ± 0.35 ^{a}
α-TERP	1.00 ± 0.00 ^{c}	0.32 ± 0.13 ^{c}	0.32 ± 0.10 ^{c}	4.11 ± 0.36 ^{b}	7.75 ± 0.27 ^{a}
CHS	1.00 ± 0.00 ^{b}	1.02 ± 0.26 ^{b}	1.26 ± 0.19 ^{b}	2.97 ± 0.12 ^{a}	3.46 ± 0.30 ^{a}
ARF 3/4	1.00 ± 0.00 ^{d}	0.40 ± 0.07 ^{e}	6.87 ± 0.68 ^{b}	8.98 ± 0.25 ^{a}	4.52 ± 0.38 ^{c}

Different letters within each row indicate significant differences ($p < 0.05$), according to ANOVA, followed by Duncan's method (regular letters) or Kruskal–Wallis one-way ANOVA on ranks, followed by the Student-Newman-Keuls post hoc test (italic letters).

Table S3. Effect of light of different spectral compositions on antioxidant capacity, phenolic compound, total terpenoids and main photosynthetic pigments content in previous-year needles.

Previous-year needles	Control	WL	RL	FRL	RL+FRL
TEAC, μmol Trolox/g FW	555.1 ± 63.5 a	532.8 ± 46.9 a	490.1 ± 16.1 a	563.4 ± 38.6 a	469.2 ± 41.9 a
GAE, mg/g FW	22.2 ± 2.1 *a*	21.1 ± 0.8 *a*	21.8 ± 0.6 *a*	24.5 ± 2.1 *a*	20.1 ± 0.9 *a*
Flavonoids, mg catechin/g FW	11.3 ± 1.5 a	10.7 ± 0.5 a	10.2 ± 0.6 a	11.5 ± 0.7 a	9.8 ± 0.6 a
Catechins+proantocyanidins, mg catechin/g FW	30.9 ± 3.9 a	33.9 ± 3.2 a	33.1 ± 1.9 a	35.0 ± 1.8 a	33.0 ± 2.3 a
Proanthocyanidins, mg cyaniding/g FW	11.1 ± 0.9 a	12.4 ± 0.8 a	11.7 ± 0.4 a	12.4 ± 0.3 a	11.5 ± 0.4 a
Terpenoids, mg/g DW	5.96 ± 0.36 a	6.45 ± 0.18 a	6.25 ± 0.47 a	7.12 ± 0.29 a	6.06 ± 0.27 a
Chl *a*, mg/g DW	2.66 ± 0.12 a	2.79 ± 0.24 a	2.96 ± 0.13 a	2.92 ± 0.29 a	2.78 ± 0.14 a
Chl *b*, mg/g DW	1.23 ± 0.06 a	1.27 ± 0.11 a	1.33 ± 0.08 a	1.55 ± 0.17 a	1.37 ± 0.09 a
Carotenoids, mg/g DW	0.54 ± 0.02 a	0.57 ± 0.05 a	0.61 ± 0.03 a	0.65 ± 0.06 a	0.58 ± 0.03 a
Chl *a*/Chl *b*	2.16 ± 0.02 a	2.19 ± 0.02 a	2.24 ± 0.04 a	1.89 ± 0.03 c	2.04 ± 0.04 b
Carotenoids/chlorophylls *a+b* ratio	0.139 ± 0.001 b	0.139 ± 0.001 b	0.143 ± 0.001 ab	0.145 ± 0.002 a	0.141 ± 0.002 ab

Different letters within each row indicate significant differences ($p < 0.05$) according to ANOVA on ranks followed by Duncan's Multiple Range post hoc test (regular letters) or Kruskal–Wallis one-way ANOVA on ranks followed by Student-Newman–Keuls post hoc test (italic letters). TEAC, Trolox equivalent antioxidant capacity; GAE, gallic acid equivalents.

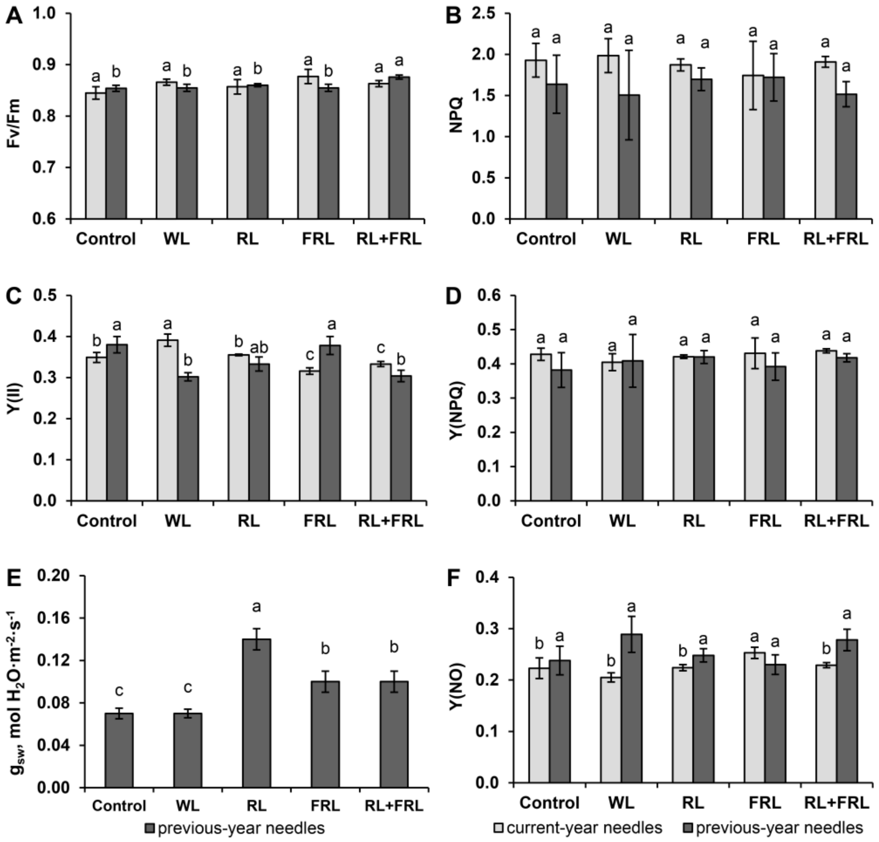

Figure S1. Effect of additional light on stomatal conductance (g_{sw}, mol H_2O $m^{-2}s^{-1}$) (**E**) and the main parameters of chlorophyll *a* fluorescence: F_v/F_m (the maximum quantum yield of PSII) (**A**); Y(II) (PSII effective quantum yield) (**C**); NPQ (non-photochemical fluorescence quenching) (**B**); Y(NO) (quantum yield of non-regulated non-photochemical energy dissipation in PSII) (**F**), Y(NPQ) (quantum yield of regulated non-photochemical energy dissipation in PSII) (**D**) in the previous-year and current-year needles of Scots pine.
